# Structure-Activity Relationship of Flavonoids Active Against Lard Oil Oxidation Based on Quantum Chemical Analysis

**DOI:** 10.3390/molecules14010046

**Published:** 2008-12-23

**Authors:** Ji-Guo Yang, Ben-Guo Liu, Gui-Zhao Liang, Zheng-Xiang Ning

**Affiliations:** 1 College of Light Industry and Food Science, South China University of Technology, Guangzhou 510640, P. R. China; E-mail: yjgscut@263.net (J-G. Y.); 2 School of Food Science, Henan Institute of Science and Technology, Xinxiang 453003, P. R. China; 3 College of Bioengineering, Chongqing University, Chongqing 400044, P. R. China; E-mail: gzliang@cqu.edu.cn (G-Z. L.); 4 College of Light Industry and Food Science, South China University of Technology, Guangzhou 453003, P. R. China; E-mail: fezhning@scut.edu.cn (Z-X. N)

**Keywords:** Flavonoid, Structure-Activity Relationship, Quantum Chemistry, Antioxidant.

## Abstract

In this study, the antioxidant activities of 15 flavonoids against lard oil oxidation were determined by using the Rancimat test. Quercetin, dihydromyricetin, luteolin and kaempferol showed the strongest antioxidant activity, with protection factor values (PF) of 11.50, 11.29, 4.24 and 2.49, respectively. The role of conjugated hydroxyl groups of the B and C ring is discussed. By using the following descriptors: *ΔH_f_* (the difference in heat of formation between flavonoids and their free radicals resulted after hydrogen atom donation) and H_BC_ (the number of conjugated hydroxyl groups of the B and C ring), the result obtained in the antioxidant Rancimat test could be successfully explained.

## Introduction

A number of studies have shown that natural antioxidants from plant sources can effectively inhibit oxidation in food and reduce the risk of age-dependent diseases. Flavonoids, widespread in fruits, vegetables, teas and medicinal plants, have received the greatest attention, and have been studied extensively, since they are highly effective antioxidants, less toxic than synthetic antioxidants such as butylated hydroxyanisole (BHA) or butylated hydroxytoluene (BHT) [1,2]. It was found that many factors influence the antioxidant activity, such as the position of OH groups, the properties of substituent groups, and hydrogen bond formation. Theoretical calculations are helpful to investigate the activity differences of antioxidants. In fact, structure-activity relationship of flavonoids as antioxidants has been explained successfully by theoretical calculations [3,4]. However, it should be pointed out that most of the studies were obtained in the simple and hydrophilic tests as DPPH radical scavenging assay [5,6]. So it is necessary to determine whether the theoretical methods are still effective to characterize the antioxidant activity of flavonoids in a complex and hydrophobic system, e.g., in the experiments that show how antioxidants protect lipid. Therefore the goal of this paper is to measure the antioxidant activity of 15 natural flavonoids in lard oil by a Rancimat test and to present the relationships between molecular structure-derived parameters and the antioxidant activity of flavonoids.

## Results and Discussion

Hydrogen-donation was thought to be the main mechanism of acton of phenolic antioxidants in oil, and the difference in heat of formation between the phenolic antioxidant and its free radical produced after H-abstraction (*ΔH_f_*), appeared to be a good index for measuring the scavenging activity of antioxidants [7]. The effectiveness of *ΔH_f_* can be understood easily, as it represents the strength of the O-H bond. The lower strength of the O-H bond corresponds to a higher scavenging activity. AM1 was selected because it was better than other semiempirical methods, such as Modified Neglect of Diatomic Differential Overlap (MNDO) and Parametric Method 3 (PM3), to calculate *H_f_* [8]. The *ΔH_f_* values and the most active hydroxyl groups of 15 flavonoids were shown in Table 1, Table 2 and Table 3.

It could be found through the analysis of these data that:

(1) In flavonoids with a C2 -C3 double bond and a C-3 hydroxyl group, the most active hydroxyl groups for H-donating are the ones attached to C4’ or C3.
(2) In flavonoids with a C2 -C3 double bond, but without a C-3 hydroxyl group, the most active hydroxyl groups for H-donating are the ones attached to C4’.
(3) In flavonoids with a C2 -C3 single bond and a hydroxyl group linked to C3 or not, the most active H-donating hydroxyl groups are the ones attached to C4’.
(4) In flavonoids with an *ortho*-dihydroxyl group on B ring, the Δ*H_f_* values of these adjacent hydroxyl groups correspond to the values of the most active hydroxyl group for hydrogen-donating. This indicates that the substitution enhances radical scavenging ability.
(5) The Δ*H_f_* value of the C-5 and C-7 hydroxyl group is significantly higher than that of the hydroxyl group on B ring. This agrees with the conclusion that C ring deactivates A ring, reported by Zhang [8].

The above analysis fully showed that considering from the antioxidation mechanism, the hydroxyl groups on B ring of flavonoids make a great contribution to the flavonoid antioxidant activity, in certain circumstances, the hydroxyl groups on C ring also contributed. Therefore, a new descriptor, the number of conjugated hydroxyl groups of the B and C ring (H_BC_), was put forward in this study. 

**Table 1:** Chemical structures, descriptors and antioxidant activity of some flavones and flavonols. 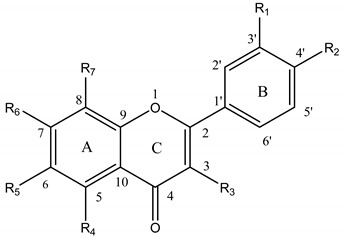

No	Flavonoid	R1	R2	R3	R4	R5	R6	R7	Δ*H_f_* (kcal/mol)	Most Active OH	H_BC_	PF
1	luteolin	OH	OH	H	OH	H	OH	H	22.09	4’-OH	2	4.24
2	kaempferol	H	OH	OH	OH	H	OH	H	21.29	3-OH	2	2.49
3	nobiletin	OCH_3_	OCH_3_	H	OCH_3_	OCH_3_	OCH_3_	OCH_3_	—	—	0	1.04
4	chrysin	H	H	H	OH	H	OH	H	30.92	7-OH	0	0.98
5	quercetin	OH	OH	OH	OH	H	OH	H	20.84	4’-OH	3	11.50
6	apigenin	H	OH	H	OH	H	OH	H	26.19	4’-OH	1	0.99
7	tangeretin	H	OCH_3_	H	OCH_3_	OCH_3_	OCH_3_	OCH_3_	—	—	0	0.99
8	camellianin A	H	OH	H	O-[rham-6-O-acetyl –glu]	H	OH	H	25.84	4’-OH	1	1.01

**Figure 1:**
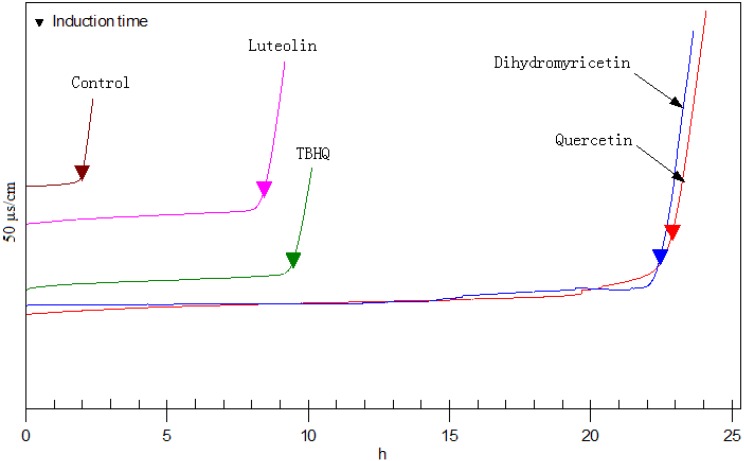
Antioxidant activity of luteolin, dihydromyricetin, quercetin and TBHQ in the Rancimat test.

In the Rancimat method, the sample is exposed to a stream of air at temperatures ranging from 50-220 °C. The volatile oxidation products (chiefly formic acid) are transferred to a measuring vessel by the air stream and absorbed in the measuring solution (distilled water). When the conductivity of this measuring solution is recorded continuously an oxidation curve is obtained, whose point of inflection is known as the induction time; this provides a good characteristic value for the oxidation stability. The protection factor values (PF) of 15 flavonoids were shown in Table 1, Table 2 and Table 3. As shown in Figure 1, quercetin, dihydromyricetin, luteolin and kaempferol showed the strongest antioxidant activity. The performance of quercetin (PF, 11.5) and dihydromyricetin (PF, 11.29) were superior to that of TBHQ (PF, 4.75).

By using H_BC_ and *ΔH_f_*, the results of this study could be successfully explained as follows:

(1) Flavonoids with H_BC_ of 0, such as chrysin, formononetin, sophoricoside, tangeretin and nobiletin, failed to show any antioxidant activity against lard oil oxidation. This is attributable to the fact that they could not easily donate hydrogen atoms.
(2) Flavonoids with H_BC_ of 1, such as genistein, naringenin, daidzein, apigenin, hesperetin and camellianin A, showed the poor antioxidant activity, with PFs of 1.11±0.01.
(3) Flavonoids with H_BC_ of 2, such as luteolin and kaempferol, showed strong antioxidant activity. The PF values of luteolin and kaempferol were 4.24 and 2.49. The performance of luteolin was superior to that of kaempferol, as the former has a structure containing *ortho*-diphenolic hydroxyl groups.
(4) Flavonoids with H_BC_ of 3, such as quercetin and dihydromyricetin, showed the strongest antioxidant activity, with PF values of 11.50 and 11.29, respectively. The Δ*H_f_* value of quercetin (20.84 kcal/mol) was less than that of dihyromyricetin (23.14 kcal/mol), which meant the capacity of H-donating of quercetin was stronger than that of dihydromyricetin. As a result, the antioxidant performance of quercetin was superior to that of dihydromyricetin.

**Table 2:** Chemical structures, descriptors and antioxidant activity of some flavanones and flavanonols 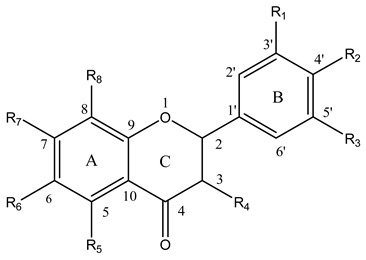

No	Flavonoid	R1	R2	R3	R4	R5	R6	R7	R8	Δ*H_f_* (kcal/mol)	Most Active OH	H_BC_	PF
1	naringenin	H	OH	H	H	OH	H	OH	H	27.49	4’-OH	1	1.09
2	hesperetin	OH	OCH_3_	H	H	OH	H	OH	H	23.15	3’-OH	1	1.28
3	dihydromyricetin	OH	OH	OH	OH	OH	H	OH	H	23.14	4’-OH	3	11.29

**Table 3:** Chemical structures, descriptors and antioxidant activity of some isoflavones 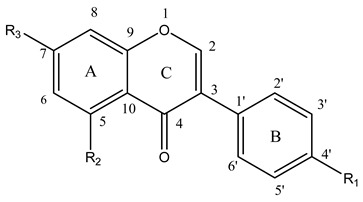

No	Flavonoid	R1	R2	R3	Δ*H_f_* (kcal/mol)	Most Active OH	H_BC_	PF
1	genistein	OH	OH	OH	24.65	4’-OH	1	1.13
2	sophoricoside	O-glu	OH	OH	49.71	5 -OH	0	0.99
3	daidzein	OH	H	OH	24.78	4’-OH	1	1.14
4	formononetin	OCH_3_	H	OH	29.79	7 -OH	0	1

In conclusion, flavonoids inhibit oil oxidation by H-donation. The antioxidant performance of flavonoids could be explained by both *ΔH_f_* and the number of hydroxyl groups in the conjugated B and C ring system.

## Experimental

### Materials and Chemicals

Apigenin and quercetin were purchased from the Chinese National Institute for the Control of Pharmaceutical and Biological Products (Beijing, P.R. China). The following flavonoids were purchased from Shanxi Huike Botanical Development Co., LTD (Xi’an, P.R. China) and their purities (about 98 %) were confirmed by HPLC: luteolin, kaempferol, nobiletin, chrysin, tangeretin, naringenin, hesperetin, genistein, sophoricoside, daidzein, and formononetin. Camellian A and dihydromyricetin with purity of 99 % came from our previous studies [9,10]. Other chemicals were of analytical grade and were used as received.

### Rancimat Test

The antioxidant activities of the samples were performed on a 743 Rancimat analyzer according to the method of Proestos *et al*. [11]. Samples of lard oil (3 g) containing flavonoids (1 μM) were subjected to oxidation at 110 °C (air flow 20 L/h). Tertiary butylhydroquinone (TBHQ) was used as the positive control. A control containing ethanol instead of flavonoids or TBHQ was also used. Induction periods (IP, h), were recorded automatically. The protection factors (PF) were calculated according to the following formula: (PF = IP_sample_/IP_control_). 

### Calculation of ΔH_f_

The heat of formation differences between flavonoids and their free radicals produced after H-abstraction (*ΔH_f_*) were calculated from computational calculations on the 3D molecular structure using the program Hyperchem 6.0 (HyperCube, Inc., Gainesville, FL, USA). The structures were drawn as 2D structures and the Molecular Mechanics force field (MM^+^) was selected for geometry optimization using the Polak-Ribiere algorithm with a maximum cycle (450) and a convergence limit of 0.1 kcal/mol. The obtained three-dimensional geometries were submitted to a final geometry optimization by the AM1 method using the Polak-Ribiere algorithm with a maximum cycle (450) and a convergence limit of 0.1 kcal/mol. Heat of formation of the parent molecules (*H_f__m_*) and the free radicals (*H_f__f_*) were obtained to calculate the index: *ΔH_f_*= H_f_*_f_* – *H_f__m_*. To make a comparison, parent molecules and free radicals were all calculated in Unrestricted Hartree Fock (UHF) approximation. In the calculations, only the most stable conformations of flavonoids and their free radicals were taken into consideration. And the corresponding hydroxyl group donating H in the most stable free radical was determined as most active hydroxyl group in a flavonoid molecule.
